# Supplementary material for: Spawning-Induced pH Increase Activates Sperm Attraction and Fertilization Abilities in Eggs of the Ascidian, Phallusia philippinensis and Ciona intestinalis
Source: Int J Mol Sci. 2023 Jan 31;24(3):2666. doi: 10.3390/ijms24032666 (PMC9917126; doi:10.3390/ijms24032666)
Supplement: Supplementary file 1 [file ijms-24-02666-s001.zip › ijms-2157535-supplementary.pdf]

Spawning-Induced pH Increase Activates Sperm Attraction and Fertilization Abilities in Eggs of the Ascidian, *Phallusia philippinensis* and *Ciona intestinalis*

Noburu Sensui [1], Yosinori Itoh [1], Nobuhiko Okura [2], Kogiku Shiba [3], Shoji A. Baba [4], Kazuo Inaba [3] and Manabu Yoshida [5,*]

Supplemental Data

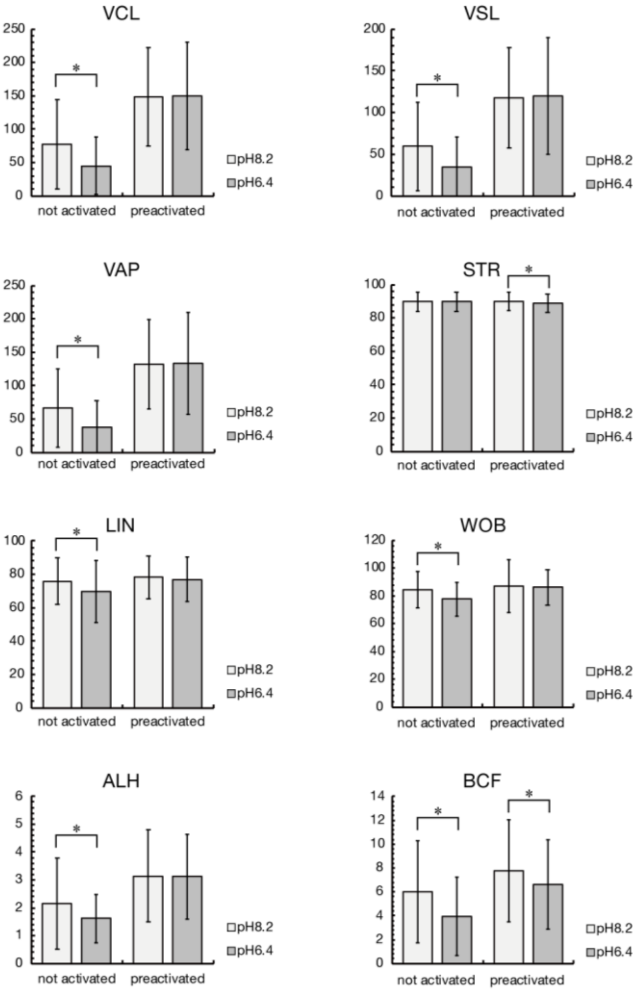

Figure S1. Motility parameters of *C. intestinalis* sperm samples used in Fig. 1C. Values are expressed as the mean ± SD of four experiments. Asterisks indicate statistical significance ($p < 0.05$; Student's t-test). Sperm movement parameters: VCL, curvilinear velocity (μm/s); VSL, straight-line velocity (μm/s); VAP, averaged path velocity (μm/s); STR, straightness; LIN, linearity; WOB, wobbling, ALH, amplitude of lateral head displacement (μm); BCF, beat/cross frequency (beats/s).

For more information on the individual movement parameters, please refer to the following reference: Verstegen, J. et al. Theriogenology 57:149-179 (2002).

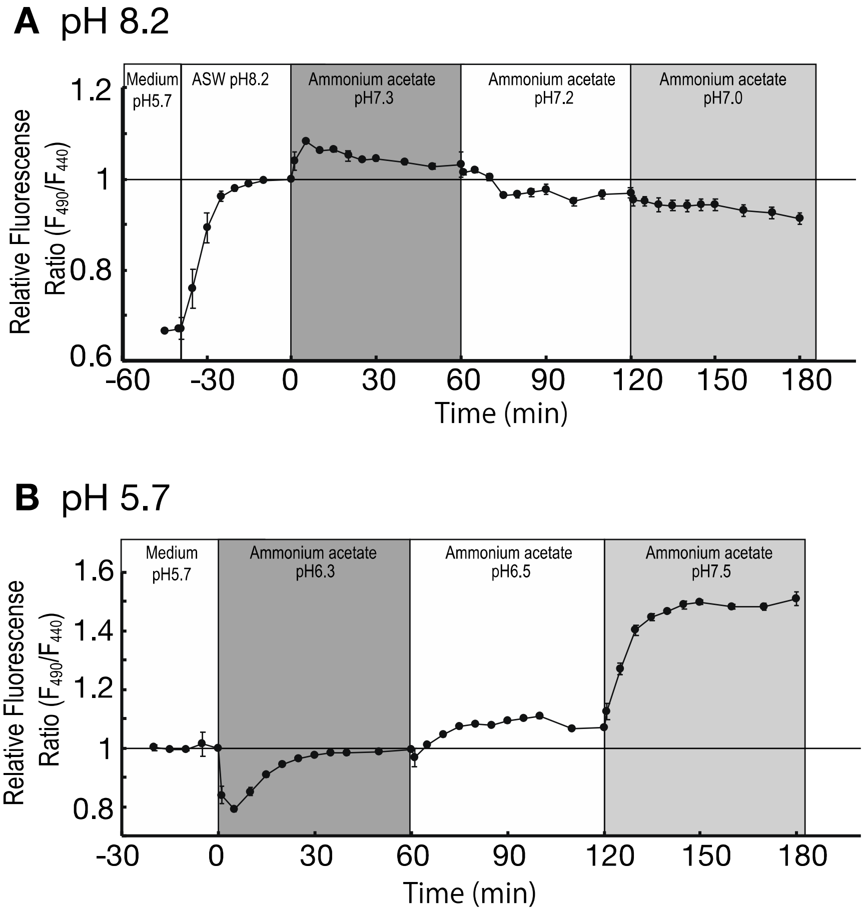

Figure S2. Estimation of intracellular pH in the *P. philippinesis* egg. (A) The egg incubated in ASW (pH8.2) for 45 min. The pH of the egg was estimated as between pH 7.2 and 7.3. (B) The egg placed in the measuring medium (pH5.7). The pH of the egg was estimated as between pH 6.3 and 6.5. Intracellular pH changes in an egg were analyzed using BCECF and evaluated using ratio of fluorescence intensities at 440 nm (F_{440}) and 490 nm (F_{490}). The difference in extracellular and intracellular pH of the egg was cancelled by replacing the ammonium acetate media, and the intracellular pH was forcibly adjusted by changing the pH in the medium. Intracellular pH of the egg was estimated by the comparison of the BCECF fluorescence between in the evaluating medium and the ammonium acetate medium. The ammonium acetate medium contains 20 mM ammonium acetate buffered with 10 mM MES (pH 6.3, pH6.5), 10 mM PIPES (pH 7.0), or 10 mM HEPES (pH 7.2, pH7.3, pH7.5). Values are expressed as mean ± SD of 4 eggs. The medium around eggs is indicated by the box in the graph.
